# Alternate Care Sites: A Novel Concept in Nepal During Disaster and Public Health Emergencies

**DOI:** 10.31729/jnma.8698

**Published:** 2024-08-31

**Authors:** Amit Kumar Singh, Shrijana Singh

**Affiliations:** 1 Department of Surgery, Nepal National Hospital, Kalanki, Kathmandu, Nepal

**Keywords:** *alternate care site*, *disaster preparedness*, *emergency response*, *hospital disaster preparedness and response plan*, *Nepal*

## Abstract

Nepal has a health care system that is complicated by access, affordability, and availability. The geographical difficulty makes the health care reach to public more difficult. Nepal being a disaster-prone country, it makes very important to strengthen the disaster preparedness up to the mark before the disaster strikes. During disaster and public health emergencies, the healthcare system quickly becomes overwhelmed or structural damage makes it non-functional, which necessitate the prior identification of Alternate Care Site by health service providers. Incorporating the identification of Alternate Care Sites into the Hospital Disaster Preparedness Plan represents a crucial transition from theoretical discussion to practical implementation for strengthening healthcare facilities. This paper discusses the concept of Alternate Care site and its implementation in Nepal's health emergency preparedness for disaster and public health emergencies.

## INTRODUCTION

"Alternate Care Site (ACS)" or "Surge Facilities", are pre-identified structure or space that allow for necessary health response when hospitals are overwhelmed or structurally damaged.^1^ The identified ACS can be used by the incoming Emergency Medical Team (EMT) - national/international, for response during disaster and public health emergencies. The commitment of identifying ACS by the health facilities pays real dividends when disaster or public health emergencies occur.^1^ In this viewpoint, we have elaborated the need of ACS and its incorporation in the Hospital Disaster Preparedness and Response Plan (HDPRP) of all health facilities for efficient response during disaster and public health emergencies.

## WHY HAVE AN ALTERNATE CARE SITE?

During 2015 earthquake(EQ) in Nepal, 1209 health facilities were fully damaged, 8773 (4843 Female) people lost their life, 22304 people were injured, 8 million people were affected and over 2.8 million people were displaced.^2^ The health system faced unprecedented strain. Due to lack of preparedness to response in situation where health facilities are damaged, rescue and treatment was badly affected. Thirty-four countries provided 18 military and 16 non military Foreign Medical Teams (4521 Personnel) to response the situation, which was not as efficient as expected due lack of identified ACS to conduct the response activities in EQ affected areas.^3,4^

Recent COVID-19 pandemic highlighted the importance of having pre-identified Alternate Care Site (ACS). The treating facilities were quickly overwhelmed which resulted in decrease treatment quality in all levels of government in Nepal.^5^ The use of ACS by China and USA during COVID-19 pandemic has proven to be effective during collapse of health facilities.^1^ The use of buildings of convenience in Nepal for isolation, treatment and management during COVID-19 pandemic in Nepal has a major contribution to the successful response to COVID-19 pandemic.

Nepal is among the most disaster-prone countries in the world and is ranked 11^th^ in EQ vulnerability and 30^th^ in flood and landslide. Unplanned settlements, population growth, weak public service provision and infrastructure, lack of regulatory standards, low literacy rates and poverty makes Nepal highly vulnerable to natural hazards.^6^ Being such vulnerable country, Ministry of Health and Population - (MoHP) Nepal has prioritized the prevention and preparedness activities in Nepal. The government has encouraged the hospitals to prepare the Hospital Disaster Preparedness and Response Plan. The inclusion of ACS in the HDPRP will allow hospitals to flex the facilities during functional or structural collapse of the hospital.

## HOW CAN THE ACS BE USED IN THE HEALTH CARE SYSTEM OF NEPAL?

Nepal has been implementing various programs to mitigate, prepare, respond to and recover from disasters. Ministry of Health and Population Nepal has identified the Hub and Satellite network of hospitals in Nepal to strengthen the coordination and communication during disaster. This network has collaborated to the successful response during Nepal's 2015 EQ and COVID-19 pandemic. The government has identified 25 Hub hospitals and over 9000 Satellite hospitals to make a network where each hub hospitals makes a sub network with the satellite hospitals.^7^ The Ministry of Health and Population -Nepal has been advocating the hospitals to have their HDPRP prepared, updated regularly and drills conducted to test the plans.

This is high time, to incorporate the concept of ACS selection in the HDPRP of all the hospitals, first in 25 Hub hospitals and then to all Satellite hospitals.

## WHICH SITE/FACILITIES CAN BE SELECTED AS ACS?

### 1. Building of Convenience:

Selection of facilities which can be used as ACS varies on the type and magnitude of disaster. Globally, the most commonly used are the "building of convenience" which are nearby non-medical structures which can be adopted as an ACS.^1,8^ Similar concept was used during COVID-19 pandemic in Nepal as well. These building of convenience can be used for isolation or triaging of the patients, however in the context of Nepal, equipping such structures to provide the response (acute or chronic) will be a problem as those hospitals will not have capacity to convert those buildings as ACS with provision of health service delivery. Each hospital can identify one building of convenience as their ACS in the HDPRP.

### 2. Nearby Health Facility:

In context of Nepal, the nearby health centre can be identified as ACS so that if a health facility is damaged or non-functional, the service provision can be extended from the nearby health centre. Each hospital can identify a nearby health centre as their ACS in the HDPRP.

### 3. Open space:

Though the geographical complexity and urbanization of Nepal doesn't favour the availability of the open spaces nearby the health care facilities, these spaces can be used as ACS for the Hospital to extend their facilities or host an incoming EMT to provide services. Thus, each hospital can identify a open space nearby as their ACS in the HDPRP.

## HOW CAN THE ACS BE USED?

The use of ACS varies with the site, type of disaster and the magnitude of the disaster. The possible uses of the ACS include the following:

- **Building of Convenience:**
  - Facility for isolation or quarantine
  - Vaccination clinic
  - Primary triage area
- **Nearby Health Facility:**
  - Acute care
  - Inpatient facility
  - Ambulatory care
  - Maternal and Child Health Care
  - Vaccination clinic
  - Essential Health Services
- **Open Space:**
  - Primary triage area
  - Establishment of temporary field hospitals
  - Space to host incoming EMT

## WAY FORWARD

Incorporating Alternate Care Sites into Nepal's Hospital Disaster Preparedness and Response Plan is essential for enhancing the country's health system resilience during disasters. Identifying buildings of convenience, nearby health facilities, and open spaces as ACS ensures continuous health care delivery when primary facilities are compromised. This proactive approach significantly strengthens Nepal's ability to respond effectively to emergencies, safeguarding public health in times of crisis.
